# Advanced age and apnea-hypopnea index predict subclinical atherosclerosis in patients with obstructive sleep apnea syndrome

**DOI:** 10.1186/2049-6958-8-9

**Published:** 2013-02-06

**Authors:** Bilal Arik, Mehmet Fatih Inci, Cesur Gumus, Kenan Varol, Meltem Refiker Ege, Omer Tamer Dogan, Ali Zorlu

**Affiliations:** 1 Department of Radiology, Cumhuriyet University Medical School, Sivas, Turkey; 2 Department of Radiology, Sütçü Imam University Medical School, Kahramanmaras, Turkey; 3 Department of Cardiology, Kavaklidere Umut Hospital, Ankara, Turkey; 4 Department of Chest Diseases, Cumhuriyet University Medical School, Sivas, Turkey; 5 Depatment of Cardiology, Private Malatya Hospital, Malatya, Turkey

**Keywords:** Age, Apnea hypopnea index, Coronary calcium score, Obstructive sleep apnea syndrome, Subclinical atherosclerosis

## Abstract

**Background:**

Both obstructive sleep apnea syndrome (OSAS) and coronary artery calcification (CAC) are considered to be related with the presence of coronary artery disease (CAD). In this study we evaluate the association between OSAS and presence of subclinical atherosclerosis assessed by tomographic coronary calcium score in patients who had OSAS but no history of known CAD.

**Methods:**

Seventy-three patients who were asymptomatic for CAD and had suspected OSAS were referred to overnight attended polysomnography. Patients were classified into 4 groups according to the Apnea-Hypopnea Index (AHI). All patients underwent computed tomographic examination for tomographic coronary calcification scoring. Physical examination, sleep study recordings, complete blood count and serum biochemistry were obtained from all patients.

**Results:**

In the whole group, AHI levels were weakly correlated with coronary calcium score (r = 0.342, p = 0.003) and body mass index (r = 0.337, p = 0.004), moderately correlated with basal oxygen saturation (r = −0.734, p < 0.001), and strongly correlated with oxygen desaturation index (r = 0.844, p < 0.001). In an univariate analysis, age, AHI, basal oxygen saturation, and oxygen desaturation index were associated with CAC in patients with OSAS. In a multiple logistic regression model, age (OR 1.108,%95 CI 1.031-1.191, p = 0.005) and AHI (OR 1.036,% 95 CI 1.003-1.070, p = 0.033) were only independent predictors of CAC in patients with OSAS with a sensitivity of 88.9% and 77.8% and a specificity of 54.3% and 56.5% respectively.

**Conclusions:**

Our findings suggest that in patients with moderate or severe OSAS and advanced age, physicians should be alert for the presence of subclinical atherosclerosis.

## Background

Obstructive sleep apnea syndrome (OSAS) is a respiratory disorder of sleep characterized by recurrent episodes of complete or partial airway obstruction, associated with intermittent arterial oxygen desaturation. The repetitive nocturnal hypoxemia experienced by patients with OSAS is associated with activation of a number of neural, humoral, thrombotic, metabolic, and inflammatory disease mechanisms, all of which play a role in the pathophysiology of cardiac and vascular diseases [1-3]. Moreover, several etiological factors in OSAS overlap with those of cardiovascular diseases making it difficult to distinguish the direct cardiovascular consequences of OSAS from its role in exacerbating concomitant cardiovascular disease [4,5]. OSAS is also associated with obesity, hypertension and metabolic dysregulation which themselves contribute to endothelial dysfunction [6,7]. In recent studies [8,9] the association of OSAS and coronary artery disease (CAD) has been documented.

Coronary artery calcification (CAC) has been shown to be highly specific for atherosclerosis and coronary artery calcium measurements have been revealed as a tool for risk stratification [10]. In a study utilizing tomographic coronary calcium score conducted by Detrano et al., it was suggested that the presence and extent of CAC is strongly correlated with the overall atherosclerotic plaque burden and with the development of coronary events [11].

In the present study we evaluate the association between OSAS and presence of subclinical atherosclerosis assessed by tomographic coronary calcium scoring in patients who had been referred to polysomnographic examination for clinically suspected OSAS and had no history of known CAD.

## Methods

### Study population

The study included 85 consecutive patients who were referred to polysomnography for clinically suspected OSAS. Patients with previous history of CAD diagnosed by coronary angiography, with the diagnosis of recent acute coronary syndrome (within the last 2 months), those with unstable angina or suspected cardiac ischemia on non-invasive tests, those with acute pulmonary infection, congenital heart disease, cardiac rhythm other than sinus, chronic renal failure, and insufficient clinical data were excluded. Finally, 73 patients (43 males, 30 females, mean age 50 ± 10 years) , who were asymptomatic for CAD, had suspected OSAS, and had undergone computed tomographic examination for tomographic coronary calcification scoring, were prospectively enrolled.

The following data also were recorded at hospitalization: age, gender, body mass index, atherosclerotic risk factors such as hypertension, diabetes mellitus, hyperlipidemia, and smoking habit, alcohol consumption and current medications. Smoking was defined as current cigarette smoking, and abstinence ≤ 2 years. Hypertension was defined as blood pressure ≥ 140/90 mmHg on two or more measurements or being on antihypertensive medication. Diabetes mellitus was defined as fasting glucose ≥ 126 mg/dl and/ or using an antidiabetic medication. Hyperlipidemia was defined as fasting total serum cholesterol more than 200 mg/dl and/or being on oral lipid-lowering agent. Physical examination, sleep study recordings, complete blood count and serum biochemistry were obtained from all patients.

Written informed consent was obtained from all patients and the study was approved by the Ethics Committee.

### Polysomnography

Patients with suspected OSAS were referred to overnight attended polysomnography(Somnostar 4100; SensorMedics Co., Yorba Linda, CA) with continuous recordings including electroencephalography, electrooculography, submental and leg electromyography, electrocardiography, tracheal sounds, noninvasive sensors for nasal or oral airflow, thoracic and abdominal respiratory movement and oxyhemoglobin level (Sat-Trak finger-pulse oximeter; SensorMedics Co). Sleep stages were manually scored using the methods of Rechtschaffen et al. [12]. Apneas were defined as a complete cessation of airflow lasting ≥ 10 seconds, and hypopnea was defined as reduction of ≥ 50% in airflow from the baseline value lasting ≥ 10 seconds and associated with 3% desaturation or an arousal. The Apnea-Hypopnea Index (AHI) was established as the ratio of the number of episodes of apnea and hypopnea per hour of sleep. Patients with AHI < 5 were included in the simple snoring group (Group 1). OSAS was defined if AHI was ≥ 5 events/ hour. Mild OSAS was defined as AHI 5 to 15/h (Group 2), moderate OSAS was defined as AHI 16 to 29/h (Group 3) and severe OSAS was defined as AHI ≥ 30/h (Group 4). The oxygen desaturation index was defined as the number of events in which oxygen saturation falls below 90% per hour of sleep [13-15]. The oxygen desaturation index, basal oxygen saturation and average oxygen saturation values were also recorded.

### Cardiac multislice computed tomography (MSCT) analysis

Computed tomography examinations were performed with an unenhanced, electrocardiogram gated, MSCT scanner (Philips Brilliance 16 Slice CT Scanner, Holland) set at a 0.75 mm section thickness with a gantry rotation time of 330 msec and a kernel value of B25f. Total coronary artery calcium score was calculated by the Agatston method as the sum of lesion scores from the four major coronary arteries (left main, left anterior descending, left circumflex, and right coronary arteries) [16].

### Statistical analysis

Parametric data were expressed as mean ± standard deviation or range (min-max), and categorical data as percentages. SPSS 17.0 (SPSS, Inc., Chicago, Illinois) was used to perform statistical procedures. Independent parameters were compared by Independent sample’s *t* test, and if there was no normal distribution, by Mann Whitney *U* test. Categorical data were evaluated by chi square test as appropriate. Correlations were evaluated by Spearman correlation test. Receiver operator characteristic (ROC) curve analysis was performed to identify the optimal cut-off point of AHI and age (at which sensitivity and specificity would be maximal) for the prediction of identify CAC. Areas under the curve (AUC) were calculated as measures of the accuracy of the tests. We compared the AUC with use of the Z test. Univariate analysis was used to quantify the association of variables with coronary artery calcification. Variables found to be statistically significant with univariate analysis were used in a multiple logistic regression model with forward stepwise method in order to determine the independent predictors of CAC in patients with OSAS. A p value < 0.05 was accepted as statistically significant.

## Results

A total of 73 patients who were referred to polysomnography for clinically suspected OSAS were evaluated. Out of these patients, 43 (58.9%) were male and 30 (41.1%) were female. The mean age was 50 ± 10 years. The mean AHI, basal oxygen saturation, oxygen desaturation index and coronary calcium score were found as 22.8 ± 19.8 (1–95), 81 ± 14%, 6.8 ± 8.3 (0–47) and 28.6 ± 87.8 (0–480), respectively (Table 1).

**Table 1:** Baseline characteristics of the patients

	**All patients (n = 73)**
Age, years	50 ± 10
Male/Female	43/30
Diabetes mellitus, n (%)	21 (29%)
Hypertension, n (%)	27 (37%)
Smoking, n (%)	38 (52%)
Body mass index, kg/m^2^	30.1 ± 5.2
Hyperlipidemia, n (%)	21 (29%)
Apnea-hypopnea index (min-max)	22.8 ± 19.8 (1–95)
Basal oxygen saturation,%	81 ± 14
Oxygen desaturation index (min-max)	6.8 ± 8.3 (0–47)
Coronary calcium score (min-max)	28.6 ± 87.8 (0–480)
Presence of coronary artery calcification, n (%)	27 (37%)

**Figure 1:**
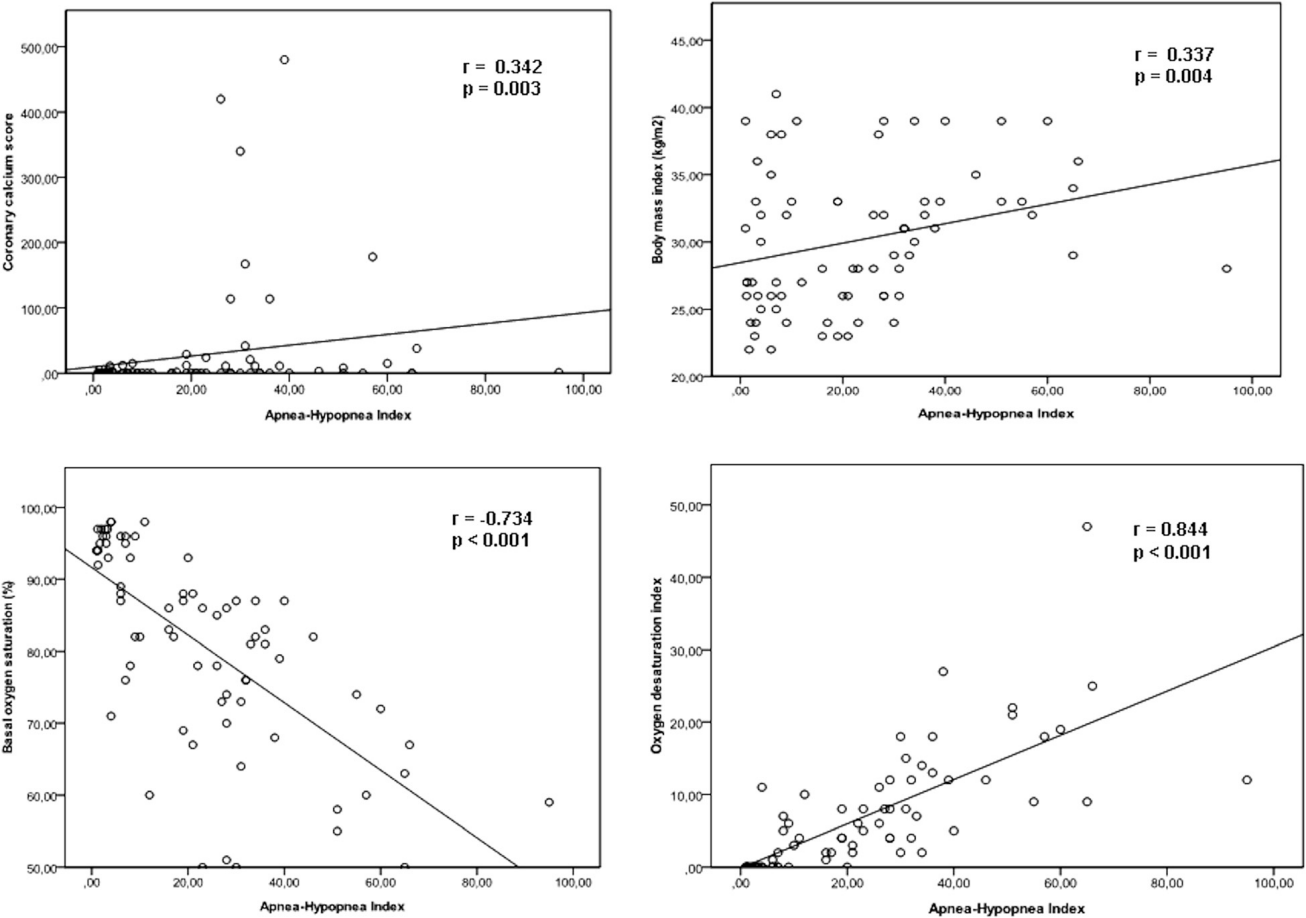
Correlation coefficients for apnea-hypopnea index.

The demographic characteristics and risk factors of patients classified according to the severity of OSAS are represented in Table 2. There were no significant differences between groups regarding age, gender, prevalence of diabetes mellitus, hypertension, hyperlipidemia and smoking. There were significant differences between the groups regarding body mass index, basal oxygen saturation, oxygen desaturation index, AHI, presence of coronary artery calcification and coronary calcium score. AHI levels were weakly correlated with coronary calcium score (r = 0.342, p = 0.003) and body mass index (r = 0.337, p = 0.004), moderately correlated with basal oxygen saturation (r = −0.734, p < 0.001), and strongly correlated with oxygen desaturation index (r = 0.844, p < 0.001) (Figure 1).

**Table 2:** Comparison of cardiovascular risk factors and obstructive sleep apnea related hypoxia parameters of patients according to the apnea-hypopnea index

	**Group 1**	**Group 2**	**Group 3**	**Group 4**	**P**
	**(AHI < 5)**	**(AHI 5–15)**	**(AHI 16–29)**	**(AHI ≥30)**	
	**(n = 16)**	**(n = 14)**	**(n = 19)**	**(n = 24)**	
Age, years	50 ± 10	48 ± 9	47 ± 9	52 ± 10	0.282
Male/Female	9/7	6/8	13/6	15/9	0.498
Diabetes mellitus	4 (25%)	2 (14%)	6 (32%)	9 (38%)	0.473
Hypertension	6 (38%)	5 (36%)	4 (21%)	12 (50%)	0.281
Smoking	6 (38%)	7 (50%)	11 (58%)	14 (58%)	0.568
Body mass index, kg/m2	28.2 ± 4.8	30.9 ± 6.4	28.4 ± 4.8	32.2 ± 4.1	0.035
Hyperlipidemia	6 (38%)	3 (21%)	3 (16%)	9 (38%)	0.332
Apnea-hypopnea index (min-max)	2.4 ± 1.1	8.0 ± 2.0	22.5 ± 4.3	45.3 ± 16.3	<0.001
	(1–4)	(6–12)	(16–28)	(30–95)	
Basal oxygen saturation,%	94 ± 6	87 ± 10	77 ± 12	71 ± 12	<0.001
Oxygen desaturation index (min-max)	0.7 ± 2.7	2.7 ± 3.2	5.1 ± 3.3	14.6 ± 19.7	<0.001
	(0–11)	(0–10)	(0–12)	(2–47)	
Coronary calcium score (min-max)	1.4 ± 3.0	1.9 ± 4.9	32.2 ± 97.5	59.5 ± 121.2	0.015
	(0–11)	(0–15)	(0–420)	(0–480)	
Presence of coronary artery calcification	4 (25%)	2 (14%)	7 (37%)	14 (58%)	0.032

Table 3 demonstrates the polysomnograpic findings and risk factors of patients according to the presence of coronary calcification. The patients in the group who had coronary artery calcification were older with marginally significant higher prevalence of diabetes mellitus. AHI and oxygen desaturation index were significantly higher, and the basal oxygen saturation values were significantly lower, in the group who had coronary artery calcification.

**Table 3:** Comparison of cardiovascular risk factors and obstructive sleep apnea related hypoxia parameters of patients with obstructive sleep apnea (AHI ≥ 5, n = 57) according to the presence of coronary calcium

	**Coronary artery calcification (−)**	**Coronary artery calcification (+)**	**p**
	**(n = 34)**	**(n = 23)**	
Age, years	46 ± 10	54 ± 7	0.002
Male/Female	18/16	16/7	0.327
Diabetes mellitus	7 (21%)	10 (44%)	0.064
Hypertension	12 (35%)	9 (39%)	0.988
Smoking	19 (56%)	13 (57%)	0.962
Body mass index, kg/m2	30.3 ± 5.7	31.1 ± 4.3	0.590
Hyperlipidemia	9 (27%)	6 (26%)	0.974
Apnea-hypopnea index	23.7 ± 16.5 (1–65)	35.5 ± 20.1 (1–95)	0.019
Basal oxygen saturation,%	79.5 ± 13.7	73.9 ± 11.1	0.108
Oxygen desaturation index	7.0 ± 8.9 (0–47)	10.8 ± 7.5 (0–27)	0.016

In the univariate regression analysis, age, AHI, basal oxygen saturation, and oxygen desaturation index were associated with CAC in patients with OSAS (Table 4). In the multiple logistic regression analysis, age (OR 1.108,% 95 CI 1.031-1.191, p = 0.005) and AHI (OR 1.036,% 95 CI 1.003-1.070, p = 0.033) were only independent predictors of CAC in patients with OSAS after adjustment of potential confounders (Body mass index, presence of hypertension and diabetes mellitus) and variables found to be statistically significant in univariate analysis (Table 5).

**Table 4:** Univariate predictors of coronary artery calcification in patients with OSAS

	**p**	**OR**	**(95% CI)**
Age, years	0.007	1.080	1.022-1.142
Apnea-hypopnea index	0.014	1.034	1.007-1.063
Basal oxygen saturation,%	0.026	0.959	0.924-0.995
Oxygen desaturation index	0.038	1.073	1.004-1.147
Presence of Diabetes mellitus	0.087	2.475	0.875-6.997
Presence of Hypertension	0.768	0.848	0.284-2.533
Body mass index, kg/m2	0.582	1.029	0.928-1.141

CI, Confidence interval; OR, Odds ratio.

**Table 5:** Multivariate predictors of coronary artery calcification in patients with OSAS

	**p**	**OR**	**(95% CI)**
Age, years	0.005	1.108	1.031-1.191
Apnea hypopnea index	0.033	1.036	1.003-1.070

CI, Confidence interval; OR, Odds ratio.

All the variables from Table 1 were examined and only those significant at a p < 0.05 level and those with a correlated age and apnea-hypopnea index levels (Body mass index, presence of hypertension and diabetes mellitus) are shown in univariate analysis.

The multiple logistic regression model included all univariate predictors and those with correlated age and apnea hypopnea index levels (Body mass index, presence of hypertension and diabetes mellitus).

**Figure 2:**
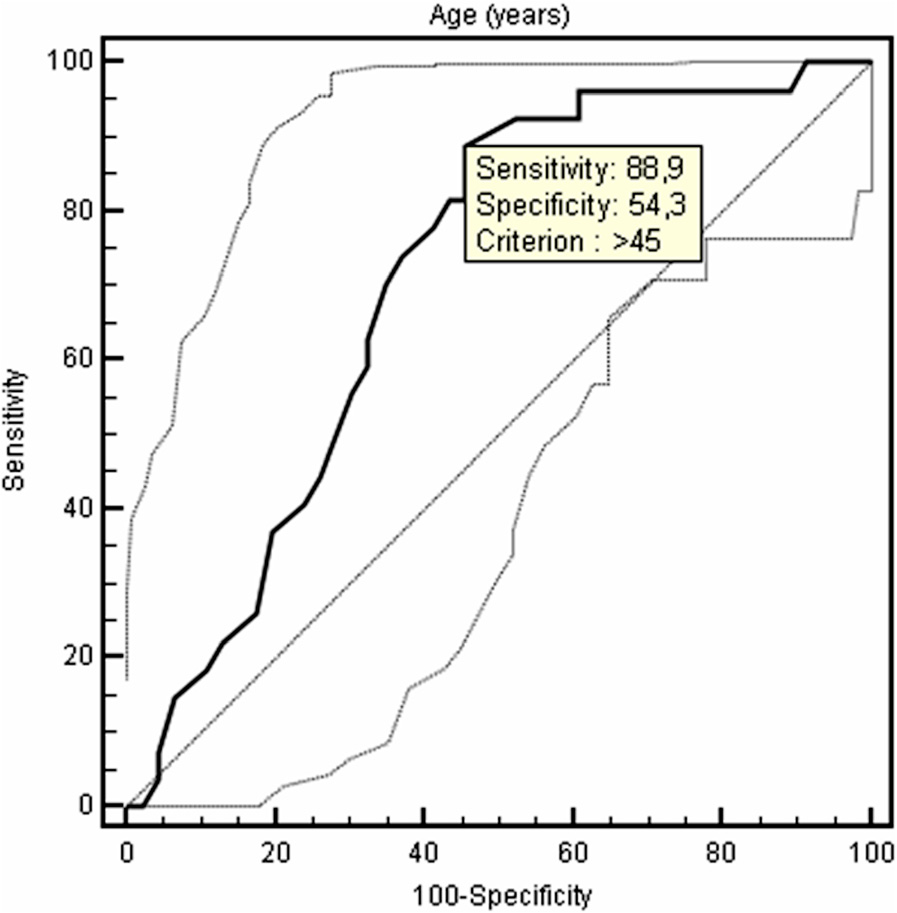
ROC curve of age for the presence of coronary artery calcification.

**Figure 3:**
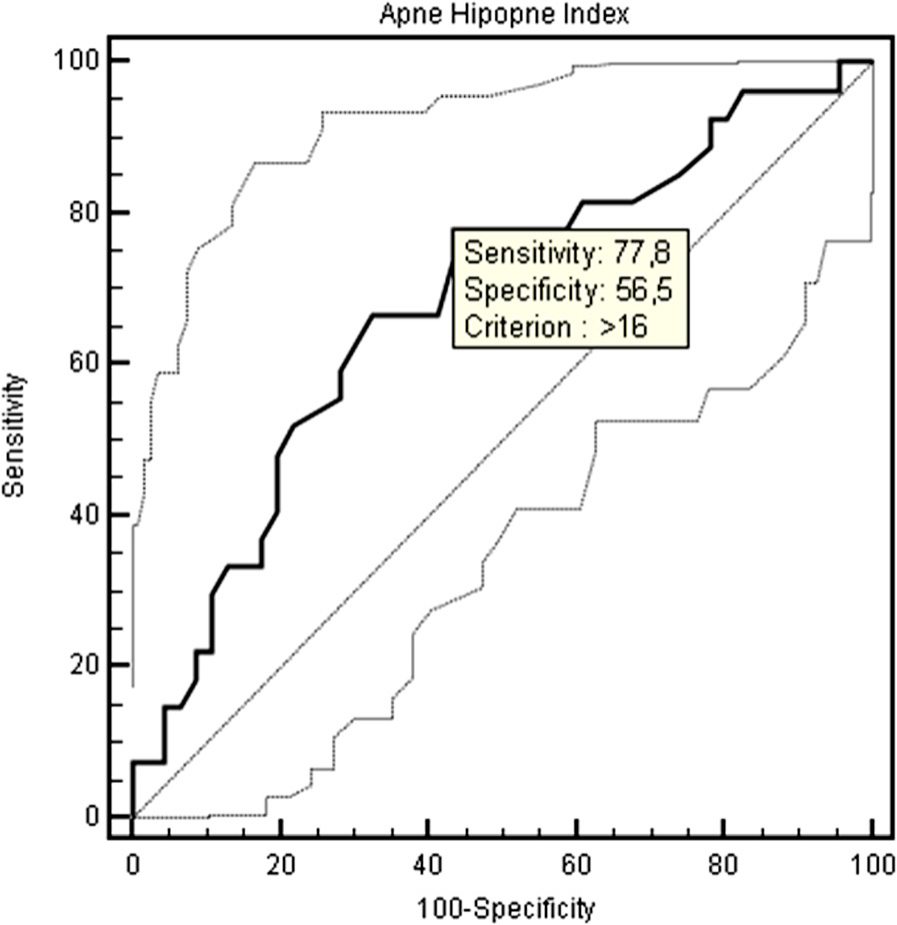
ROC curve of apnea-hypopnea index for the presence of coronary artery calcification.

**Figure 4:**
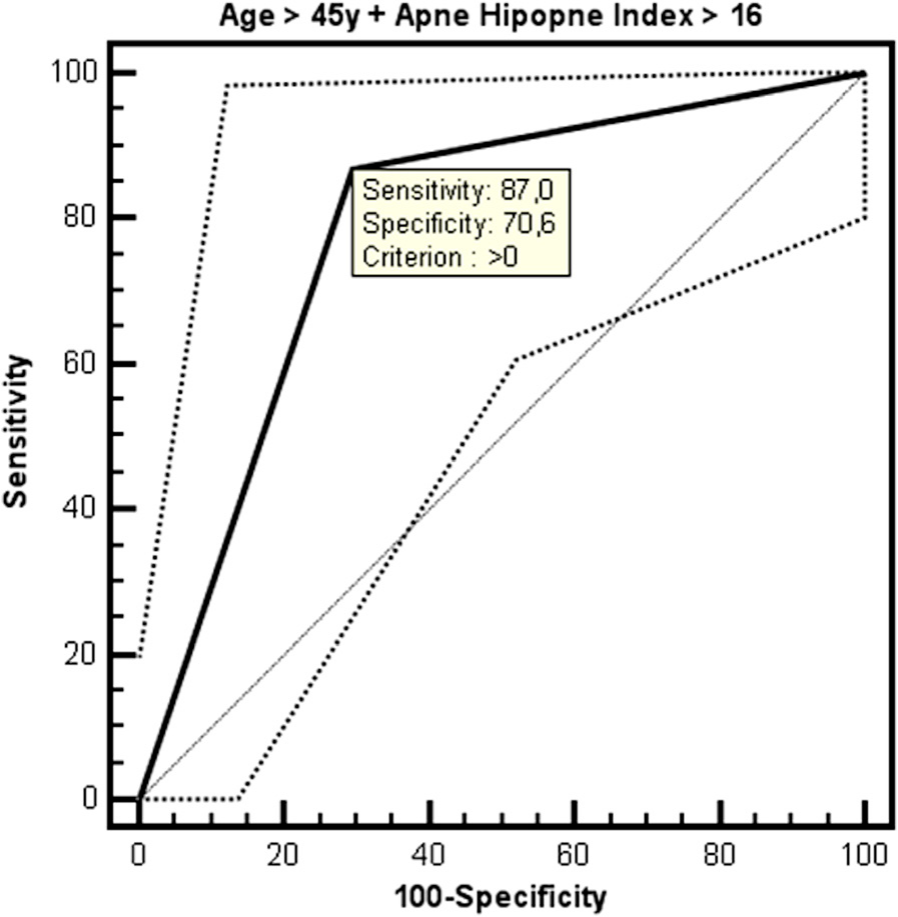
ROC curve for age > 45 years together with apnea-hypopnea index > 16 to predict the presence of coronary artery calcification.

The optimal cut-off point of age for the prediction of CAC was > 45 years with a sensitivity of 88.9% and a specificity of 54.3% (AUC: 0.711, 95% CI: 0,593 to 0,811, Figure 2). The optimal cut-off point of AHI for the prediction of CAC was > 16 with a sensitivity of 77.8% and a specificity of 56.5% (AUC: 0.682, 95% CI: 0,563 to 0,786, Figure 3). An additional ROC analysis also showed that considering the age >45 and AHI >16 together in patients with OSAS, specificity increased with unchanged sensitivity for predicting subclinical atherosclerosis (87% sensitivity, 70.6% specificity, AUC: 0.788, 95% CI: 0.665-0.910, Figure 4).

## Discussion

Our study demonstrated that age, AHI, basal oxygen saturation, oxygen desaturation index and presence of diabetes mellitus were associated with CAC in patients with OSAS. However, even after controlling these parameters, age and AHI were the only independent predictors of subclinical atherosclerosis in patients with OSAS.

Obstructive sleep apnea which occurs in approximately 9–24% of the general population [17] is an emerging cardiovascular risk factor [4,5]. The Sleep Heart Health Study showed a modest increase in the odds ratio of CAD in patients with severe OSAS compared to controls [2]. Hung et al. [18] also reported that in patients with myocardial infarction, OSAS was a strong risk factor as obesity, smoking and hypertension. In a group of 23 OSAS patients without symptoms or history of CAD, about one-third showed asymptomatic ST-segment depression during sleep, but only one patient was found to have positive exercise stress test result, suggesting a low prevalence of symptomatic CAD in OSAS [19]. Therefore determination of subclinical atherosclerosis in patients with OSAS can be important in order to prevent the adverse cardiovascular events.

Although in recent studies [20,21] increased cardiovascular morbidity have been confirmed in patients with OSAS, the occurrence of other cardiovascular risk factors often limited the assessment of an independent pathogenetic role for OSAS. Whereas in our study, although there was no difference in regard to the presence of cardiovascular risk factors, when patients were evaluated according to the severity of OSAS we found that the number of patients who had coronary calcification were higher in the group with higher AHI and the patients with higher AHI had significantly higher coronary calcium score compared to patients with lower AHI. On the other hand , when OSAS patients were evaluated according to the presence of coronary calcium, it was found that patients with coronary calcium were significantly older and had marginally significant higher prevalence of diabetes mellitus with higher AHI. Our study showed that the severity of OSAS was related to subclinical atherosclerosis irrespective of the presence of other cardiovascular risk factors except for age. Thus, it may be suggested that the presence of OSAS is a marker of subclinical atherosclerosis dependent on increasing age and the severity of subclinical atherosclerosis correlates with the severity of OSAS.

A recent study conducted by Sorajja et al. revealed that increasing severity of OSAS was related to an increase in the severity of CAC independent from other risk factors [22]. To confirm this study, our results showed that the risk of subclinical atherosclerosis was increasing with a sensitivity of 77.8% when AHI > 16 which is an indicator of moderate-to-severe OSAS. Besides, Kepez et al. observed that the relationship between the severity of OSAS and the increase in cardiovascular risk was dependent only on the age of patients [23]. On the contrary, our results demonstrate that the level of AHI and advanced age are independently associated with the development of CAC. Regardless of the severity of OSAS, patient’s age > 45 becomes an indicator of cardiovascular risk with the sensitivity of 88.9%. An additional ROC analysis also showed that considering the age > 45 and AHI > 16 together in patients with OSAS, specificity increased with unchanged sensitivity for predicting subclinical atherosclerosis (87% sensitivity, 70.6% specificity).

Although the mechanisms underlying cardiovascular disease in patients with OSAS are still poorly understood, endothelial dysfunction, oxidative stress, and inflammation are long-term consequences that mediate cardiovascular disease in patients with OSAS [24-27]. An important mechanism of atherosclerosis in OSAS is inflammation resulting in endothelial dysfunction [24]. Several mediators that have been implicated in the pathogenesis of atherosclerosis are abnormal in patients with OSAS. C-reactive protein, a marker of systemic inflammation, and endothelin-1, a potent long-acting vasoconstricting substance are shown to be elevated in OSAS [25]. Moreover, intermittent hypoxia and reperfusion during repetitive episodes of nocturnal apnea may be involved in the generation of highly reactive oxygen radicals, as well as in ischemia-reperfusion injury to the vascular wall, resulting in increased risk for atherosclerosis [26,27].

## Conclusions

In conclusion, in patients with moderate or severe OSAS and age greater than 45, physicians should be alert for the presence of subclinical atherosclerosis in order to prevent adverse cardiovascular outcomes.

## Competing interest

The authors declare that they have no conflict of interest.
